# Supplementary material for: Intrinsic timescales in the visual cortex change with selective attention and reflect spatial connectivity
Source: Nat Commun. 2023 Apr 3;14:1858. doi: 10.1038/s41467-023-37613-7 (PMC10070246; doi:10.1038/s41467-023-37613-7)
Supplement: Supplementary file 3 — Reporting Summary [file 41467_2023_37613_MOESM3_ESM.pdf]

nature portfolio

Corresponding author(s): Tatiana A. Engel, Anna Levina

Last updated by author(s): Feb 27, 2023

Reporting Summary

Nature Portfolio wishes to improve the reproducibility of the work that we publish. This form provides structure for consistency and transparency in reporting. For further information on Nature Portfolio policies, see our Editorial Policies and the Editorial Policy Checklist.

Statistics

For all statistical analyses, confirm that the following items are present in the figure legend, table legend, main text, or Methods section.

n/a	Confirmed	
☐	☒	The exact sample size (*n*) for each experimental group/condition, given as a discrete number and unit of measurement
☐	☒	A statement on whether measurements were taken from distinct samples or whether the same sample was measured repeatedly
☐	☒	The statistical test(s) used AND whether they are one- or two-sided<br>*Only common tests should be described solely by name; describe more complex techniques in the Methods section.*
☐	☒	A description of all covariates tested
☐	☒	A description of any assumptions or corrections, such as tests of normality and adjustment for multiple comparisons
☐	☒	A full description of the statistical parameters including central tendency (e.g. means) or other basic estimates (e.g. regression coefficient) AND variation (e.g. standard deviation) or associated estimates of uncertainty (e.g. confidence intervals)
☐	☒	For null hypothesis testing, the test statistic (e.g. F, t, r) with confidence intervals, effect sizes, degrees of freedom and P value noted<br>*Give P values as exact values whenever suitable.*
☐	☒	For Bayesian analysis, information on the choice of priors and Markov chain Monte Carlo settings
☒	☐	For hierarchical and complex designs, identification of the appropriate level for tests and full reporting of outcomes
☐	☒	Estimates of effect sizes (e.g. Cohen's *d*, Pearson's *r*), indicating how they were calculated

Our web collection on statistics for biologists contains articles on many of the points above.

Software and code

Policy information about availability of computer code

Data collection	Data were recorded using the Omniplex system (Plexon) in attention task 1 and the fixation task and with the Digital Lynx recording system (Neuralynx) in attention task 2.
Data analysis	Data was analyzed with a custom code written in Python 3.7 and MATLAB 2021a.

For manuscripts utilizing custom algorithms or software that are central to the research but not yet described in published literature, software must be made available to editors and reviewers. We strongly encourage code deposition in a community repository (e.g. GitHub). See the Nature Portfolio guidelines for submitting code & software for further information.

Data

Policy information about availability of data

All manuscripts must include a data availability statement. This statement should provide the following information, where applicable:

- Accession codes, unique identifiers, or web links for publicly available datasets
- A description of any restrictions on data availability
- For clinical datasets or third party data, please ensure that the statement adheres to our policy

All behavioral and electrophysiological data used in this study are available on Fighshare at https://doi.org/10.6084/m9.figshare.19077875.v1 (fixation task), https://doi.org/10.6084/m9.figshare.16934326.v3 (attention task 1) and https://doi.org/10.6084/m9.figshare.21972911.v2 (attention task 2).

Human research participants

Policy information about studies involving human research participants and Sex and Gender in Research.

Reporting on sex and gender	*Use the terms sex (biological attribute) and gender (shaped by social and cultural circumstances) carefully in order to avoid confusing both terms. Indicate if findings apply to only one sex or gender; describe whether sex and gender were considered in study design whether sex and/or gender was determined based on self-reporting or assigned and methods used. Provide in the source data disaggregated sex and gender data where this information has been collected, and consent has been obtained for sharing of individual-level data; provide overall numbers in this Reporting Summary. Please state if this information has not been collected. Report sex- and gender-based analyses where performed, justify reasons for lack of sex- and gender-based analysis.*
Population characteristics	*Describe the covariate-relevant population characteristics of the human research participants (e.g. age, genotypic information, past and current diagnosis and treatment categories). If you filled out the behavioural & social sciences study design questions and have nothing to add here, write "See above."*
Recruitment	*Describe how participants were recruited. Outline any potential self-selection bias or other biases that may be present and how these are likely to impact results.*
Ethics oversight	*Identify the organization(s) that approved the study protocol.*

Note that full information on the approval of the study protocol must also be provided in the manuscript.

Field-specific reporting

Please select the one below that is the best fit for your research. If you are not sure, read the appropriate sections before making your selection.

☒ Life sciences ☐ Behavioural & social sciences ☐ Ecological, evolutionary & environmental sciences

For a reference copy of the document with all sections, see nature.com/documents/nr-reporting-summary-flat.pdf

Life sciences study design

All studies must disclose on these points even when the disclosure is negative.

Sample size	Three male monkeys were used for the experiments in this study, which is a standard sample size for primate studies (Nandy and Reynolds, Neuron, 2017; Ferro et al., PNAS, 2021, Spyropoulos et al., PNAS, 2018). Animals' ability to perform the task determined the number of trials in each recording session. The number of simultaneously recorded neurons was determined by the properties of the linear multielectrode arrays used for neural recordings.
Data exclusions	3 out of 25 recording sessions in monkey G (AT1) were excluded due to very short trial durations that lead to large statistical biases in autocorrelations. For estimating the timescales, we excluded sessions with autocorrelations dominated by noise or strong oscillations that could not be well described with a mixture of exponential decay functions. We excluded a session if the autocorrelation fell below 0.01 in lags smaller or equal to 20 ms (for details see Methods and Supplementary Figure 11).
Replication	All analysis results were replicated in three monkeys.
Randomization	Participants were not allocated into groups. No subject randomization was implemented.
Blinding	Blinding is not relevant. There were no differences between monkeys that would conceivably create biases.

Reporting for specific materials, systems and methods

We require information from authors about some types of materials, experimental systems and methods used in many studies. Here, indicate whether each material, system or method listed is relevant to your study. If you are not sure if a list item applies to your research, read the appropriate section before selecting a response.

Materials & experimental systems

n/a	Involved in the study
☒	☐ Antibodies
☒	☐ Eukaryotic cell lines
☒	☐ Palaeontology and archaeology
☐	☒ Animals and other organisms
☒	☐ Clinical data
☒	☐ Dual use research of concern

Methods

n/a	Involved in the study
☒	☐ ChIP-seq
☒	☐ Flow cytometry
☒	☐ MRI-based neuroimaging

Animals and other research organisms

Policy information about studies involving animals; ARRIVE guidelines recommended for reporting animal research, and Sex and Gender in Research

Laboratory animals	Three male monkeys (Macaca mulatta, 6-9 years old) were used in these experiments.
Wild animals	Monkeys were obtained from a breeding facility. No wild animal were used.
Reporting on sex	Recordings were performed in three male macaque monkeys. This sample size is too small to make any meaningful statements about the effect of sex on the mechanisms that we found. In the literature, basic motor- and decision-related findings tend to replicate between sexes. In particular, a study performed on both sexes found a lack of evidence for sex differences in higher cognitive functions in macaques (Nagy, bioRxiv, 2017). Since our dataset is similar in nature, differences between sexes are unlikely to skew our scientific findings.
Field-collected samples	No field collected samples were used in this study.
Ethics oversight	All experimental procedures for the fixation task and attention task 1 were in accordance with NIH Guide for the Care and Use of Laboratory Animals, the Society for Neuroscience Guidelines and Policies, and Stanford University Animal Care and Use Committee. All experimental procedures for the attention task 2 were in accordance with the European Communities Council Directive RL 2010/63/EC, and Use of Animals for Experimental Procedures, and the UK Animals Scientific Procedures Act.

Note that full information on the approval of the study protocol must also be provided in the manuscript.
